# Cotton domestication: dramatic changes in a single cell

**DOI:** 10.1186/1741-7007-8-137

**Published:** 2010-11-15

**Authors:** Briana L Gross, Jared L Strasburg

**Affiliations:** 1 USDA-ARS, National Center for Genetic Resource Preservation, 1111 S. Mason Street, Fort Collins, CO 80521, USA; 2 Department of Biology, Indiana University, 915 E. 3rd Street, Myers #150, Bloomington, IN 47405, USA

## Abstract

Investigations on the nature of genetic changes underpinning plant domestication have begun to shed light on the evolutionary history of crops and can guide improvements to modern cultivars. A recent study focused on cotton fiber cells tracks the dramatic genome-wide changes in gene expression during development that have accompanied selection for increased fiber yield and quality.

See Research article: http://www.biomedcentral.com/1741-7007/8/139

## Domestication for fiber

Domesticated plants serve as excellent models for studying evolutionary processes, most notably for understanding the genetic effects of strong selection. This is true because in crops both the target (agronomic traits such as increased resource allocation to fruit or seed size, or loss of dispersal mechanisms) and mechanism (human action) of selection are often known, in contrast to wild systems where our understanding of selective pressures is often incomplete. Much progress has been made towards identifying the genetic outcomes of artificial selection, using techniques that include artificial domestication experiments, archaeobotany, population genetics, quantitative genetics, and molecular genetics. One of the first domestication genes was cloned in 1997 (*tb1*, which affects plant architecture and inflorescence structure in maize) [1], and now multiple plant domestication and improvement genes are identified each year [2]. Nonetheless, much progress remains to be made, specifically in terms of understanding how these individual genetic changes (discovered in a necessarily reductionist framework) interact and affect the organism as a whole. In this issue, a paper by Rapp *et al*. [3] tackles the question of how broad-scale gene expression can change with domestication by contrasting patterns of gene expression in developing fiber cells from domesticated and wild cotton.

**Figure 1:**
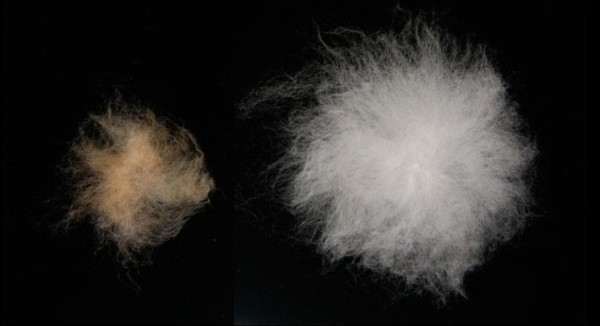
**Seed and associated fiber from a wild and domestic cotton plant**. The seed on the left is from the wild cotton plant *Gossypium hirsutum *var. *yucatanense*; on the right is a seed from the domesticated *G. hirsutum*.

Cotton is one of the most important crops not primarily farmed as a human or animal food (although one of the byproducts is an edible oil); instead it is grown mainly for the fiber it produces (actually elongated and thickened seed trichomes, or epidermal cells) and is the world's largest source of renewable natural textile fiber. There are two major forms of domesticated cotton, both of which originated in the New World. *Gossypium barbadense*, known as 'Pima' or 'Egyptian' cotton, was (despite its common name) domesticated in the Peruvian Andes between 4,000 and 5,000 years ago [4]. 'Upland' cotton, *Gossypium hirsutum*, makes up the bulk of the world's cotton crop, and was domesticated at approximately the same time in the Yucatan peninsula [5]. Both Pima and Upland cotton underwent many phenotypic changes during the domestication process, including reductions in seed dormancy, a shift to a more compact plant architecture, and loss of photoperiod sensitivity (that is, the plant is no longer dependent on changes in day-length to induce flowering). The major change, however, is seen in the seed trichomes that make up the cotton fiber, which have become longer, finer and stronger in the crop than in the wild form (Figure 1).

## Gene expression in Upland cotton

Rapp *et al*. investigated the dramatic morphological and developmental changes seen in cotton fibers by comparing patterns of gene expression in these cells through five developmental time points in a variety of domesticated *G. hirsutum *(cultivar TM-1) and an individual from a population of its wild relative, *G. hirsutum *var. *yucatanense*. Using a microarray designed to examine expression levels at more than 40,000 genes, Rapp *et al*. examined changes in gene expression between wild and domesticated cotton at each developmental stage as well as changes among developmental stages within each lineage. Overall, 9,465 genes, or almost a quarter of all genes surveyed, were differentially expressed in the domesticated cotton compared to wild cotton. And while both the wild and the domesticated varieties showed changes in gene expression between each developmental stage, when summed over all developmental stages more than twice as many genes (12,626 versus 5,273) showed differential expression during development in the domesticated variety.

While this many differences in gene expression is remarkable in itself, the pattern of changes was also quite striking. A cluster analysis designed to describe the most prominent general patterns of gene expression across the developmental time-series revealed eight different gene expression profiles: for example, one group of genes showed low expression early in development, a peak of higher expression at intermediate stages, and decreasing expression levels later in development. This expression pattern was seen in 1,441 and 799 genes in the wild and domesticated samples, respectively; but only 118 genes show this pattern in *both *wild and domesticated cotton. Of the 1,323 genes with this pattern in wild cotton that are differentially expressed in the domesticate, almost 70% showed a nearly opposite pattern - they were up-regulated during the two earliest developmental stages. Similarly, of the 1,159 genes that show lowest expression at the earliest developmental stage and increasing expression at each subsequent stage in wild cotton, more than half are dramatically up-regulated at the earliest developmental stages in the domesticate. In contrast, many genes with high expression early in development in the wild fiber show decreased early expression in domesticated fiber. These 'modular' changes in gene expression with respect to developmental timing suggest that large networks of genes may have been similarly affected by the selection pressures encountered during domestication. Interestingly, as suggested above, gene expression changes between wild and domesticated cotton fiber cells are most pronounced at the earliest developmental stages, implicating the early stages as critical to this aspect of cotton domestication and, potentially, further improvement.

Finally, Rapp *et al*. identified many genes of known function in cotton or *Arabidopsis *that showed changes in gene expression consistent with their function and possible role in the morphological changes between wild and domesticated cotton. For example, several genes involved in cytoskeletal function were up-regulated in the domesticated variety at some developmental stages, and may be associated with the increased fiber length and quality seen in the crop. Likewise, a number of genes associated with sucrose transport and metabolism, which is involved in cotton fiber cell elongation and cell wall synthesis, are up-regulated in the later stages of domesticated cotton fiber development. While direct functional involvement of these genes in cotton domestication will need to be confirmed through further experimental studies, these findings, along with those discussed above concerning more general patterns of gene expression changes during development, emphasize the importance of going beyond identifying genes that are globally over- or under-expressed in one sample compared to another, and instead considering the complexities of gene expression through time.

## What the future holds

A few important caveats should be kept in mind when considering these results. For example, Rapp *et al*. only examined gene expression changes in fiber cells, with a focus on understanding the genetic basis of differences in fiber cell morphology and development specifically. However, without expression data from other cell types it is not possible to determine which gene expression changes are fiber cell-specific, and which might reflect more general expression differences between wild and domesticated cotton. In addition, the gene expression changes identified here do not all reflect changes specifically induced by human selection for cotton domestication and improvement traits: further experiments are required to test the functional importance of specific genes and gene expression changes. These could include sequence characterization of the coding and regulatory regions of interesting differentially expressed genes, comparison of the genomic locations of these genes with previously identified quantitative trait loci for cotton fiber quality and other aspects of cotton domestication, and further functional characterization through transformation experiments. Finally, it will be valuable to see how these results extend to other *G. hirsutum *cultivars and wild populations, and to the independently domesticated *G. barbadense*. Results from a previous, less extensive, experiment provide tantalizing evidence that gene expression in *G. barbadense *fiber cells may not be any more dynamic than that seen in the wild form [6], possibly indicating an important difference between the domestication events.

Despite its economic importance, we still know relatively little about the genetic basis of domestication in *Gossypium*, at least in terms of the number and types of genes involved, and it remains an open question whether these extensive changes in gene expression are due to many small genetic changes or reflect the effect of changes in a few regulatory genes. The latter would, however, explain some of the modularity of the changes in expression patterns, as large, complex genetic networks may be affected by one or a few upstream changes. In the majority of crop plants explored thus far, genes of major effect appear to be responsible for an impressive proportion of the phenotypic changes between the wild and domesticated species, although the sunflower (*Helianthus annuus*) is an exception, appearing to lack major mutational leaps during domestication [7]. This question can be addressed by quantitative trait locus mapping, and mapping experiments conducted for cotton so far do indicate that there are genes with major effects on fiber traits [8], although the mapping experiments have mainly involved crosses between the two domesticates [8,9] or crosses within a domesticated species [10], rather than between a domesticate and its progenitor, which would allow direct detection of changes during domestication. The exact genetic basis for the expression patterns seen by Rapp *et al*. in *G. hirsutum*, and whether they are distinct from those seen in *G barbadense*, must await further investigation.
